# Nitrogen and Carbon Nitride-Doped TiO_2_ for Multiple Catalysis and Its Antimicrobial Activity

**DOI:** 10.1186/s11671-021-03573-4

**Published:** 2021-07-26

**Authors:** Atif Ashfaq, Muhammad Ikram, Ali Haider, Anwar Ul-Hamid, Iram Shahzadi, Junaid Haider

**Affiliations:** 1 Solar Cell Application Research Lab, Department of Physics, Government College University Lahore, Lahore, Punjab 54000 Pakistan; 2 Department of Clinical Medicine and Surgery, University of Veterinary and Animal Sciences, Lahore, Punjab 54000 Pakistan; 3 Core Research Facilities, King Fahd University of Petroleum and Minerals, Dhahran, 31261 Saudi Arabia; 4 Punjab University College of Pharmacy, University of the Punjab, Lahore, 54000 Pakistan; 5 Tianjin Institute of Industrial Biotechnology, Chinese Academy of Sciences, Tianjin, 300308 China

**Keywords:** Co-precipitation, Photocatalysis, Sonocatalysis, Dye degradation, Methylene blue

## Abstract

Nitrogen (N) and carbon nitride (C_3_N_4_)-doped TiO_2_ nanostructures were prepared using co-precipitation route. Fixed amount of N and various concentrations (0.1, 0.2, 0.3 wt%) of C_3_N_4_ were doped in TiO_2_ lattice. Through multiple techniques, structural, chemical, optical and morphological properties of samples were thoroughly investigated. XRD results verified anatase TiO_2_ presence along the substitutional doping of N, while higher degree of crystallinity as well as increased crystallite size were noticed after doping. HR-TEM study revealed formation of nanostructures incorporated on two dimensional (2D) C_3_N_4_ nanosheet surface. Elemental composition was checked out using EDS technique which confirmed the presence of dopant in product. Optical characteristics were evaluated with UV–vis spectroscopy which depicted representative redshift in absorption spectra resulted in a reduction in bandgap energy in N/C_3_N_4_-doped TiO_2_ samples. The formation of Ti–O–Ti bonds and different molecular vibrations were disclosed by FTIR. Trap sites and charge carrier’s migration in the materials were evaluated with PL spectroscopy. Multiple catalytic activities (photo, sono and photo-sono) were undertaken to evaluate the dye degradation performance of prepared specimen against methylene blue and ciprofloxacin. Further, antimicrobial activity was analyzed against *Escherichia coli* (*E. coli*) and *Staphylococcus aureus* (*S. aureus*) bacteria.

## Introduction

In the past few years, researchers and scientists have paid greater attention to energy crisis and environmental and aquatic pollution. In today’s technology driven society, relentless consumption of fossil fuels is serving to make these issues worse [1]. Fossil fuels, the rich energy-generation source, are contracting worldwide and developed countries are switching to sustainable and environment-friendly technologies. On the other hand, textile industry produces wastewater containing 5–15% of untreated organic dyes. Around 1 × 10^5^ dyes are in use globally and 7 × 10^5^ ton dyestuff is generated by the textile industry worldwide annually. Discharge of these untreated dyes not only affects the oxygen and nitrogen cycle connected to photosynthesis but also causes grave esthetic deterioration [2]. These are some of the serious environmental issues that need to be addressed to sustain human society in the long term.

Properties of bulk materials depend a lot on size and structure [3]. In this regard, nano-scaled semiconductors with diverse properties are used for photocatalytic and dye degradation applications [4]. Various transition metals (TMs) Ti, Cu, Fe, Co and non-metals (NMs) N, C, S etc., exhibit distinct physical and chemical properties [5]. In this regard, a combination of TM (titanium–dioxide) and NM (nitrogen) exhibit additive properties. Combination of semiconductors (with metals and metal oxides) that bear appropriate band arrangements possess striking applications in energy production and water treatment [6]. Rare earth metals oxides such as TiO_2_, CdO, CoO, ZnO, etc., are leading candidates for many such applications. Amongst these, TiO_2_ holds limited activity in visible region due to its high band gap value (3.0 eV for rutile phase, 3.2 eV for anatase phase) [7], low surface area and high electron–hole recombination [8]. In 1972, TiO_2_ was used for the decomposition of water using UV light [9]. Since then, photocatalysis with semiconductors has gained much attention due to their potential applications such as in hydrogen production and environmental cleaning [10]. Semiconductors with unique band gap (occupied valence band and unoccupied conduction band) serve as suitable catalysts for photochemical reactions. Currently, the main focus is visible light region through band gap tuning of inorganic semiconductor.

TiO_2_ is a semiconductor with prominent features including tunable band gap, simple synthesis routes and ecological-friendly nature. Therefore, to improve the photocatalytic activity of TiO_2_ nanostructures, modulation of its band gap was undertaken by adding nitrogen (N) for better absorption of solar spectrum. N was added through CH_4_N_2_O source which contains 46% of its concentration [7, 11]. As a result, bandgap of TiO_2_ varied from 3.2 to 3.06 eV (substitutional replacement of N with oxygen) or it can also be varied from 3.2 to 2.46 eV (interstitial doping) which is more preferable for visible light [12–14]. Dopant C_3_N_4_ is a 2D material which resolves the impediment of low surface area of TiO_2_ to enhance photocatalytic activity upon addition in various concentrations and exhibit broader spectral response near the visible region compared to pristine TiO_2_. Moreover, C_3_N_4_ has promising applications due to a suitable band gap (2.7 eV) which has the ability to enhance photocatalytic activity of TiO_2_ for use in dye degradation [15–18].

TiO_2_ nanostructures can also be utilized as antibacterial agent. The antibacterial activity of TiO_2_ nanostructures is assigned to reactive oxygen species (ROS) such as hydroxyl radicals and hydrogen peroxide, which induce severe oxidative stress on bacterial strain generated under illumination. Therefore, TiO_2_ is a potential candidate for antibacterial agent. The generated ROS provide a contact between TiO_2_ and cells which kills the cell due to damage aroused in DNA and cell membrane that ultimately results in termination of cell cycle [19]. The antimicrobial activity of prepared antibiotic also depends on surface area, morphology, crystallinity, concentration/dosage, pH of the solution, capping agent, and also the nature of microorganisms. Combining the merits of C_3_N_4_ with TiO_2_ and N, prepared composite is important to solve the problems of the environmental crisis in worldwide including organic water pollutants and pathogenic microbial contaminations [20, 21]. Few studies have been reported for the antibacterial activity and degradation of different dyes [5, 8, 11], best of our knowledge, this novel study report the efficiency of C_3_N_4_-doped N/TiO_2_ nanocomposites synthesized by co-precipitation route.

In this paper, co-precipitation method was used to synthesize pristine and doped TiO_2_ nanostructures. This study revealed the strong contact formation of TiO_2_ with dopants (N and C_3_N_4_) which efficiently increased the photocatalytic activities against methylene blue and ciprofloxacin as well as antibacterial property against *E. coli* and *S. aureus* bacteria.

## Experimental Details

### Materials

**Fig. 1:**
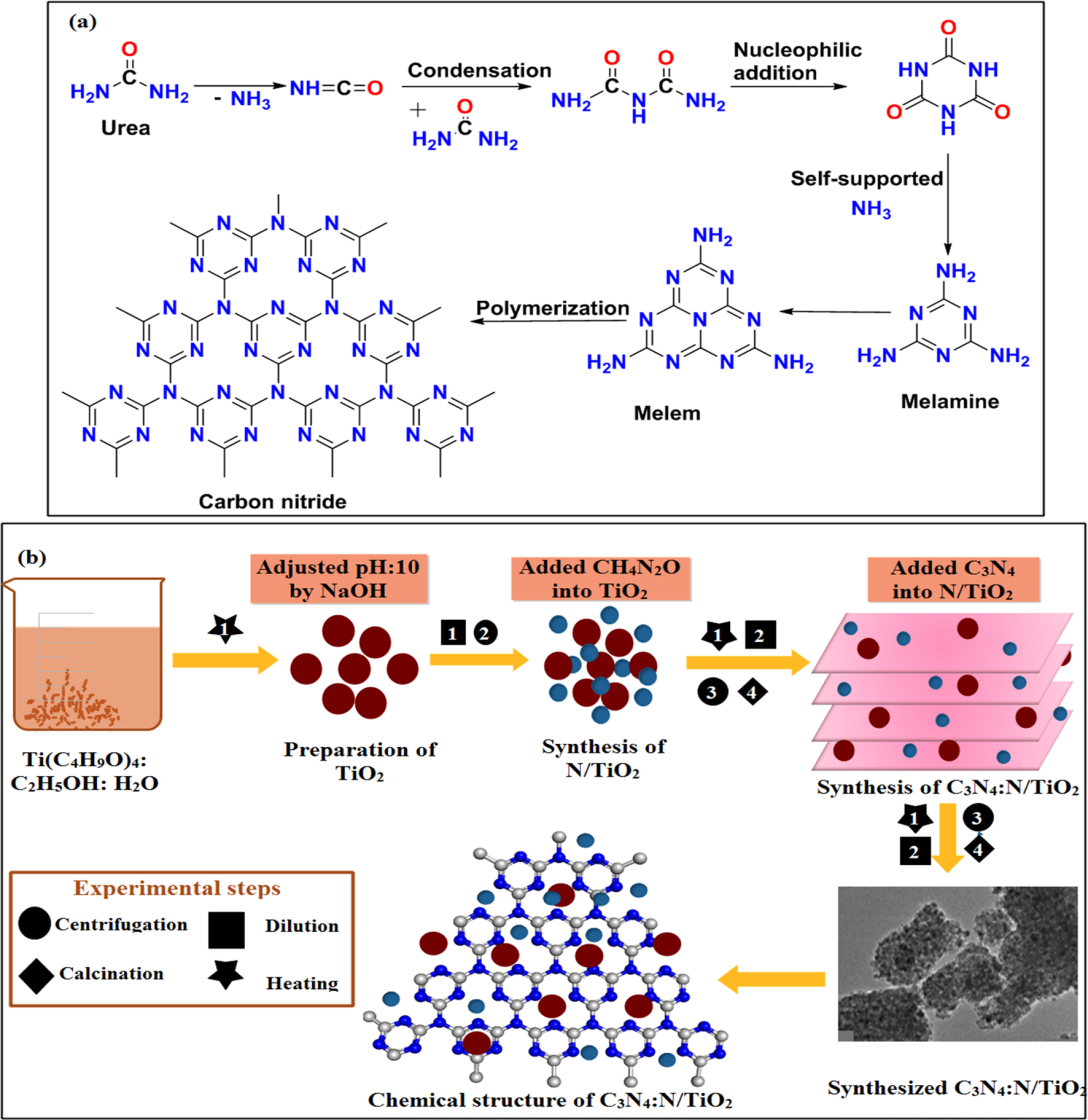
**a** Synthesis mechanism of C_3_N_4_. **b** Schematic illustration of synthesis process for C_3_N_4_:N/TiO_2_

Urea (CH_4_N_2_O) (99%) and titanium (IV) butoxide (Ti(C_4_H_9_O)_4_) (98%) were received from Sigma-Aldrich, Germany while ethanol (C_2_H_5_OH) (95%) was purchased from Panreac. Carbon nitride (C_3_N_4_) was obtained from pyrolysis of CH_4_N_2_O in the laboratory (Fig. 1a). All the reagents were used without further purification.

### Preparation of TiO_2_ Nanostructures

Titanium-dioxide (TiO_2_) was prepared by adopting co-precipitation method where 55 mL of ethanol, 13 mL of Ti(C_4_H_9_O)_4_ and 5 mL of deionized water (DI water) were mixed and stirred vigorously for 30 min. Ethanol (~ 100 mL) and DIW (~ 100 mL) were added to the stirred solution for 2 h at 50 °C. The pH of solution was maintained up to ~ 10 using NaOH (0.5 M) solution. Then, solution was centrifuged and dried at 90 °C for 10 h. After that, sample was annealed at 450 °C for 4 h to achieve stable anatase nanostructures (Fig. 1b).

### Preparation of C_3_N_4_Doped N-TiO_***2***_

Various concentrations (0.1, 0.2, 0.3 wt%) of C_3_N_4_ was doped into N-TiO_2_ mixture. Ti(C_4_H_9_O)_4_ (17.45 mL), CH_4_N_2_O (3 g), C_2_H_5_OH and DI water were added under vigorous stirring. Samples were sonicated for homogenous mixing. Sonicated samples were centrifuged at 4000 rpm and dried at 90 °C for 10 h and annealed at 450 °C for 4 h to obtain stable nanostructures. Six samples were prepared and named as TiO_2_, nitrogen-doped TiO_2_ (N-TiO_2_ as 0:1), pristine carbon nitride (C_3_N_4_ 1:0) and different concentrations of C_3_N_4_ in N-TiO_2_ named as 0.1:1, 0.2:1, 0.3:1.

### Evaluation of Photocatalytic Activity

The photocatalytic activity (PCA) of synthesized catalysts was assessed by estimating the degradation rate of a combination of two toxic dyes namely methylene blue (MB) and ciprofloxacin (CF) in aqueous solution. The stock solution of dyes was prepared in DIW (10 mg/1000 mL) and 10 mg of prepared catalyst (pristine TiO_2_, C_3_N_4_, 0:1, 0.1:1, 0.2:1, 0.3:1) was added to 50 mL stock solution. After homogeneous stirring, solution was placed in a sealed box under mercury (Hg) lamp (wavelength 400 to 700 nm and power 400 W) at ~ 15 cm distance to avoid overheating. After 20 min interval, 3 mL solution was separated to check concentration of dyes present in the solution by utilizing UV–Vis spectroscopy. The degradation efficiency was determined by the formula given as:

1 $${\text{Degradation}}\,{\text{efficiency}}\,(\% ) = \frac{Co - Ct}{{Co}} \times 100$$

where *C*_o_ and *C*_t_ initial and final concentration of dye at time *t* = 0 and at final time t, respectively [22].

### 2-Diphenyl-1-Picrylhydrazyl (DPPH) Radical Scavenging Assay

Free radical scavenging activity of all samples was examined using the method reported by Kibiti and Afolayan [23] with certain modifications. Various concentrations of pristine TiO_2_, C_3_N_4_, and C_3_N_4_ doped TiO_2_ nanoparticles (0–500 µg/mL) were prepared and mixed with equal volume of 0.1 mM DPPH solution. The reaction mixture was vortex and incubated for 30 min in dark at ambient temperature. Ascorbic acid was employed as a reference antioxidant. Absorbance of mixture was measured at 517 nm using spectrophotometer. The % scavenging ability was calculated using equation:

$${\text{DPPH}}\,{\text{scavenging}}\,{\text{rate}} \left( \% \right) = \frac{{\left( {A_{0} - A_{1} } \right)}}{{A_{0} }} \times 100$$

where *A*_0_ is absorption of control (Methanol + DPPH) and *A*_1_ is absorbance of sample.

### Bacterial Segregation and Identification

With ovine mastitic milk specimen’s antibacterial evaluation was undertaken on *S. aureus* and *E. coli* isolated after initial screening at ovine blood agar (5%) and finally on mannitol salt agar (MSA) and MacConkey agar (MCA), respectively. Coagulase, catalase, and Gram' staining protocols were used to classify extracted commodities (biochemically and morphologically).

### Antimicrobial Activity

Well diffusion procedure was adapted to assess antibacterial effects of N and C_3_N_4_ co-doped TiO_2_ by swabbing 1.5 × 10^8^ CFU mL^−1^ of purified bacterial isolates on MCA and MSA, separately. On swabbed dishes, 6 mm deep wells were drilled with aseptic well borer. Various dilutions of doped nanomaterial’s e.g., 500 and 1000 µg/50 µL were placed into wells as minimum and maximum dosage in contrast with ciprofloxacin as positive control (5 μg/50 μL) and DIW (50 μL) as negative control. The sensitivity of all prepared samples was measured with a Vernier caliper after overnight incubation (37 °C) of Petri plates. Antibacterial evaluation was contemplated by utilizing one-way analysis of variance [24].

### Material Characterization

Phase transition and crystal structure of binary-doped TiO_2_ was examined with XRD utilizing spectrum Bruker system with monochromatic Cu K-α (*λ* = 0.154 nm and 2*θ* = 10°–80°) with a scan rate of 0.05° per minute. The study of functional groups and chemical analysis was undertaken utilizing FTIR spectrometer (PerkinElmer 3100) with range of spectra from 4000 to 400 cm^−1^ in 32 scans and a resolution of 0.2 cm^−1^. The optical study was carried out with Genesys 10S spectrophotometer (ranging from 200 to 800 nm). Interlayer spacing and surface morphology of prepared products were inspected through HR-TEM and EDS spectrometer, JSM-6460LV, and HRTEM Philips CM30 and JEOL JEM 2100F. Photoluminescence spectroscopy was carried out to inquire transfer and recombination of e^−^ to h^+^ pairs utilizing a spectrofluorometer (JASCO, FP-8300).

## Results and Discussion

**Fig. 2:**
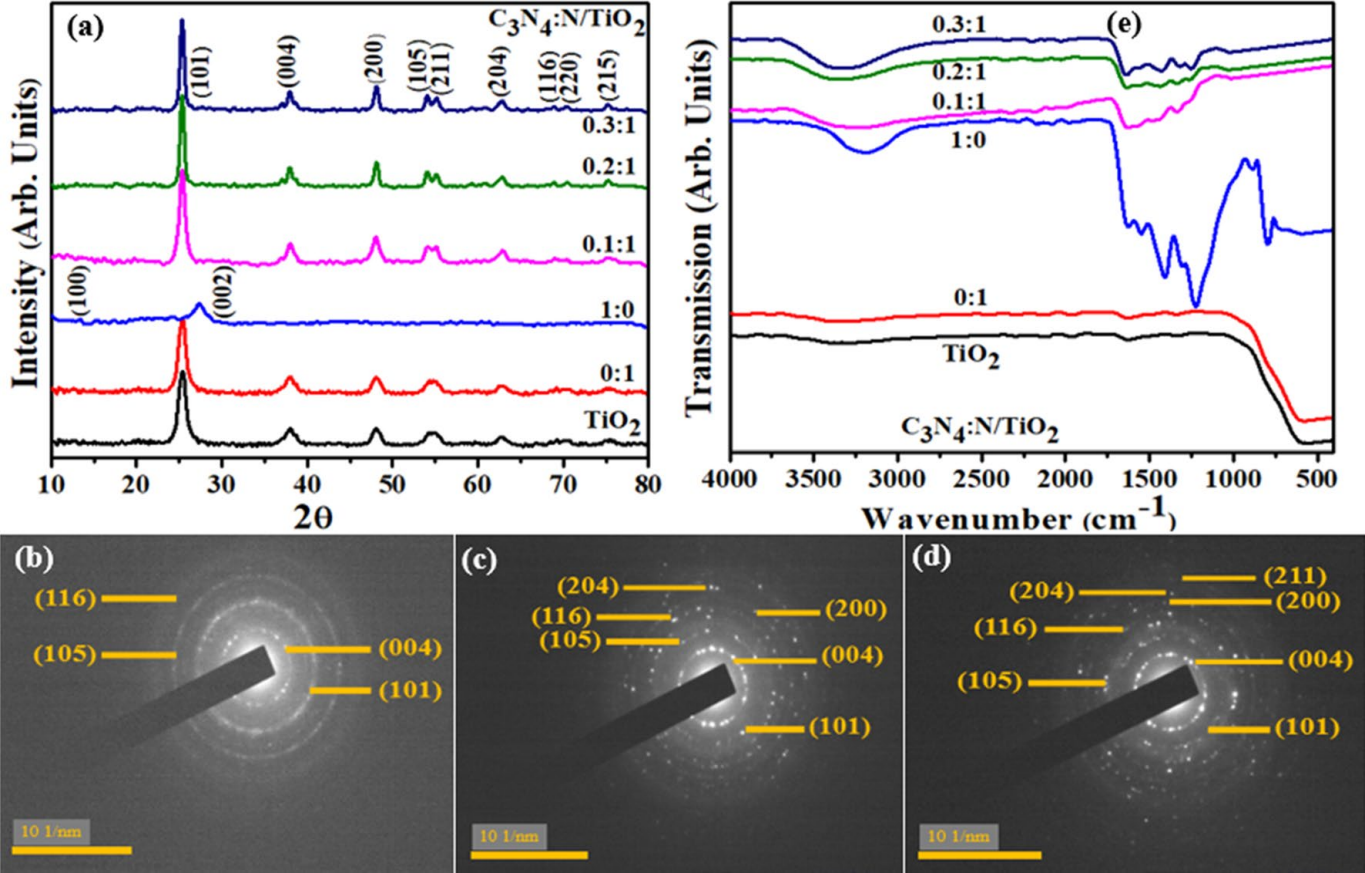
**a** XRD pattern. **b** FTIR spectra. **c**–**e** SAED profiles of as-synthesized co-doped TiO_2_. **c** TiO_2_. **d** 0:1. **e** 0.1:1

To analyze the structure of the crystal, phase purity and size of crystallites, XRD was employed on control and N/C_3_N_4_-doped TiO_2_ in 2*θ* range of 10°–80° (Fig. 2a). Acquired spectra revealed peaks at 25.4°, 37.8°, 48.1°, 53.9°, 55.1°, 62.7°, 68.6, 70.3° and 75.1° attributed to (101), (004), (200), (105), (211), (204), (116), (220), (215) planes of tetragonal anatase formation, respectively (JCPDS no. 21-1272). For N-TiO_2_ composite, no significant change in XRD spectrum was observed which might be referred to relatively lower concentration of N in the composite. Hexagonal structure for C_3_N_4_ was confirmed from peaks generated at 13.2° (100) and 27.4° (002) reflecting the standard spectrum (JCPDS no. 87–1526). No shift in peaks was observed upon doping for 0.1:1 (lower) and 0.2:1 (intermediate) and 0.3:1 (higher) concentration samples, however the sharpness in peaks indicated the successful coupling of C_3_N_4_:N/TiO_2_, resulting in enhanced crystallinity and structural quality [25, 26]. The interlayer spacing of pristine TiO_2_ (~ 0.352 nm) and N-TiO_2_ (~ 0.35 nm) were calculated from the most intense peak (101) using Debye-Scherer formula which were further verified by HR-TEM observation.

SAED (Selected Area Electron Diffraction) profiles of pristine TiO_2_, 0:1 (N-TiO_2_) and 0.1:1 sample are given in Fig. 2b–d. Images were indexed with diffraction planes (004), (101), (105), (116), (200), (204), (211) confirmed by XRD results, showing the tetragonal crystal structure of TiO_2_.

Various functional groups and chemical compositions present in as prepared samples were identified using FTIR analysis (Fig. 2e). In acquired spectra, it can be seen that absorption band stationed at 400–700 cm^−1^ corresponds to Ti–O and Ti–O–Ti stretching vibration modes, which indicated TiO_2_ formation. This vibration band has been linked with physiosorbed water protons in synthesized samples [27–29]. Band observed at about 1635 and 3200–3500 cm^−1^ referred to hydroxyl (O–H) group and physically absorbed water on pristine TiO_2_ surface, respectively [30, 31]. In C_3_N_4_ spectrum, absorption band at 1624 cm^−1^ was attributed to C-N heterocycle stretching vibrational modes [32], while four bands at 1232, 1304, 1411, 1556 cm^−1^ were referred to aromatic C–N stretching vibrational modes [33, 34]. Notably, all characteristic peaks of TiO_2_ and C_3_N_4_ can be observed, validating the formation of C_3_N_4_:N/TiO_2_ nanostructure.

**Fig. 3:**
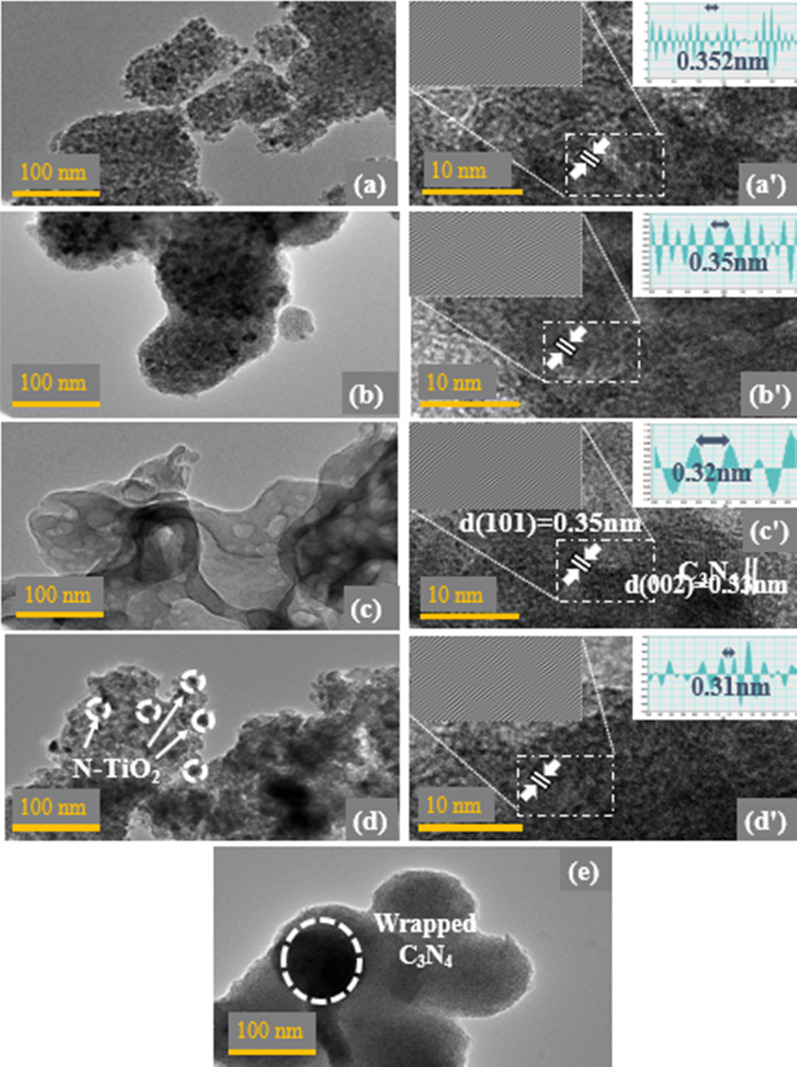
**a**–**e** HR-TEM micrographs of pristine TiO_2_, 0:1, 1:0, 0.1:1, 0.3:1, respectively. **a**′–**d**′ Interlayer spacing measured using HR-TEM images of pristine TiO_2_, 0:1, 0.1:1, 0.3:1, respectively

The morphology and crystal structure of pristine TiO_2_ (Fig. 3a), 0:1, 1:0, 0.1:1 and 0.3:1 nanostructure were studied by TEM analysis. Figure 3b represents N-TiO_2_ composite with a high surface energy that leads to aggregation [35] and Fig. 3c is the illustration of C_3_N_4_, a mesoporous nanosheet (NS). Upon C_3_N_4_ addition, N-TiO_2_ composite was embedded and well distributed on NS which also roughly confirmed the C_3_N_4_ wrapping, as illustrated in Fig. 3d. This intimate interfacial contact between N-TiO_2_ composite and NS was necessary for photocatalytic activity. Upon doping, crystallinity of prepared nanostructures improved and after higher doping, NS wrapped N-TiO_2_ composite and ultimately made an efficient interfacial contact (Fig. 3e). In HR-TEM image of 0.1:1 (Fig. 3c′), interlayer spacing was calculated to be 0.35 and 0.33 nm pertaining to (101) and (002) crystal planes of N-TiO_2_ composite and C_3_N_4_, respectively, in consistence with XRD results. Interlayer spacing has been measured for each sample using Gatan software, given in Fig. 3á–d́.

**Fig. 4:**
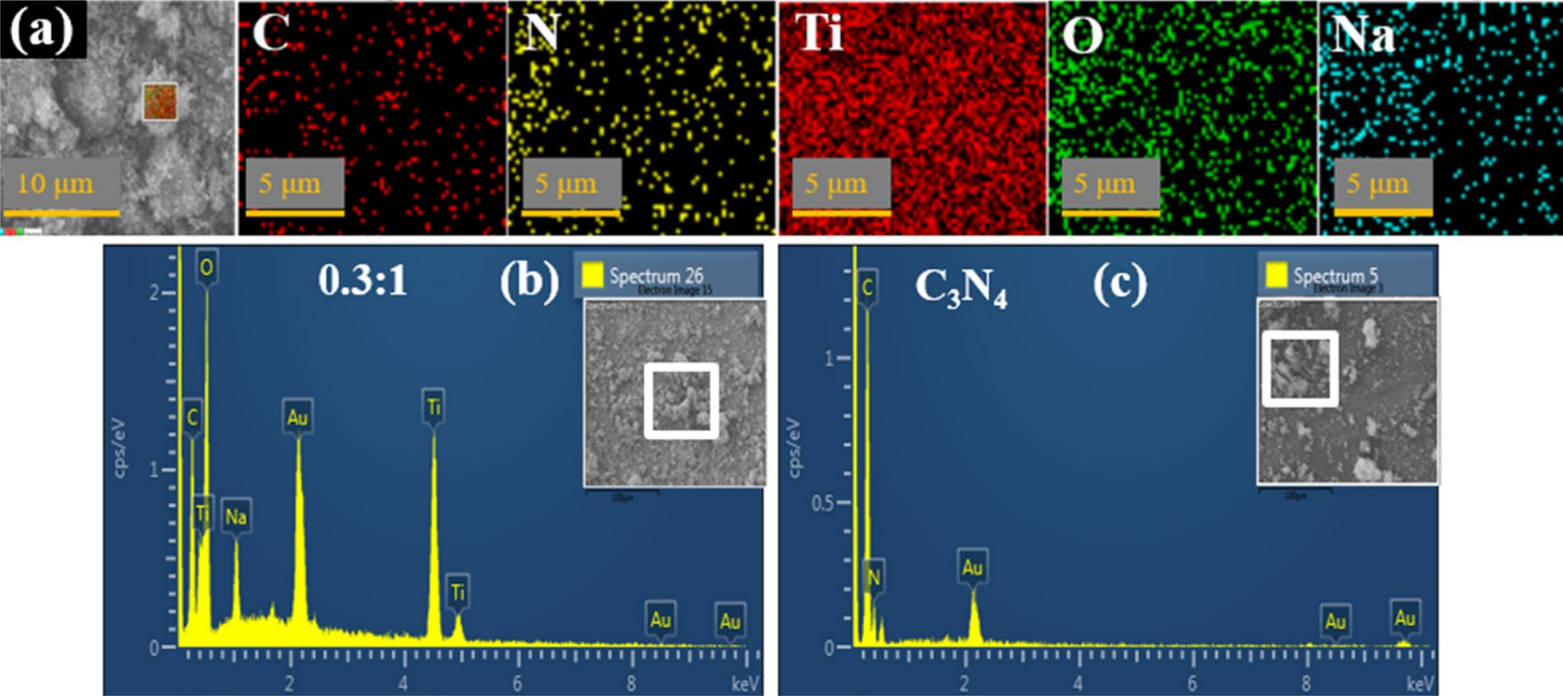
**a** Mapping of 0.3:1 sample to ensure distribution of all components (**b**–**c**) EDX images of 0.3:1 (higher doping) and 1:0, respectively

In order to check further interfacial contact, EDX mapping of as-prepared C_3_N_4_ (1:0) and 0.3:1 (higher doping) samples was conducted to inspect distribution pattern of its components. As revealed in Fig. 4a, five components (C, N, Ti, O, Na) were found to be uniformly dispersed in higher doped specimen. Sodium (Na) came from sodium hydroxide (NaOH) added for maintaining pH of solution up to ~ 10. Combined with HR-TEM and XRD results, it recommended that within 0.3:1 sample, N and TiO_2_ nanoparticles are certainly well dispersed inside wrapped C_3_N_4_ NS and indicated intimate contact as well.

Elemental composition was evaluated by EDX to confirm the purity of 0.3:1 (higher doping) and 1:0 (Fig. 4b–c). As illustrated in Fig. 4b, Ti indicated peaks at 4.5 and 4.95 keV, oxygen (O) peak at 0.5 keV, C and N peaks at 0.3 and 0.4 keV, along with several other positions were detected, confirming successful incorporation of binary-dopant species with anatase TiO_2_.

**Fig. 5:**
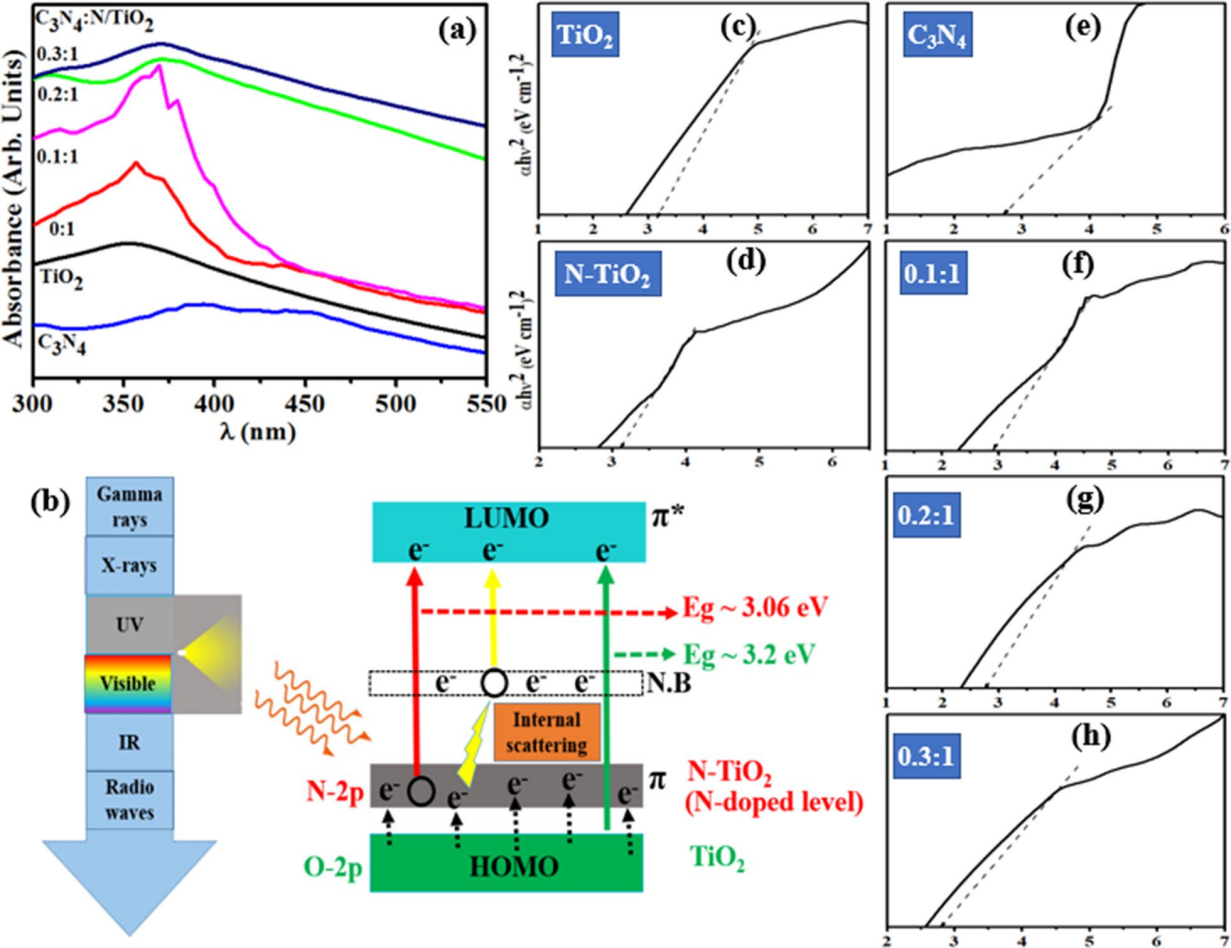
**a** Optical absorbance spectra (**b**) possible transitions and internal scattering in UV-vis spectroscopy (**c**–**h**) determination of bandgap of TiO_2_, N-TiO_2_, C_3_N_4_, 0.1:1, 0.2:1 and 0.3:1, respectively

To determine optical performance of undoped and doped TiO_2_, UV–vis spectroscopy was used in the range 300–550 nm. TiO_2_ has characteristic absorbance peak found around ~ 350 nm, with N-doping, slight redshift was observed in absorption spectra caused by overlapping of 2*p* orbitals of O_2_ and N, as shown in Fig. 5a [36]. An increase in spectral absorbance was observed upon C_3_N_4_ doping into N-TiO_2_ composite, attributed to complete planarization of C_3_N_4_ (non-overlapping of adjacent orbitals). Enhanced absorptive ability in UV-region was assigned to internal scattering and harmonious effect from N-TiO_2_ and C_3_N_4_’s *π* to π^*^ and *n* to π^*^ transitions, respectively, as depicted in Fig. 5b [37]. Peaks redshifted due to molecular engineering of C_3_N_4_ in N-TiO_2_ composite that potentially advanced absorption and transition ability of charge carriers [38]. Tuac transformation was applied to calculate bandgaps of prepared samples. For TiO_2_ bandgap was calculated to be 3.2 eV and gradual decrease in bandgap energies were observed close to Fermi level after adding N and C_3_N_4_ to ~ 2.9 eV, as given in Fig. 5c–h [39].

**Fig. 6:**
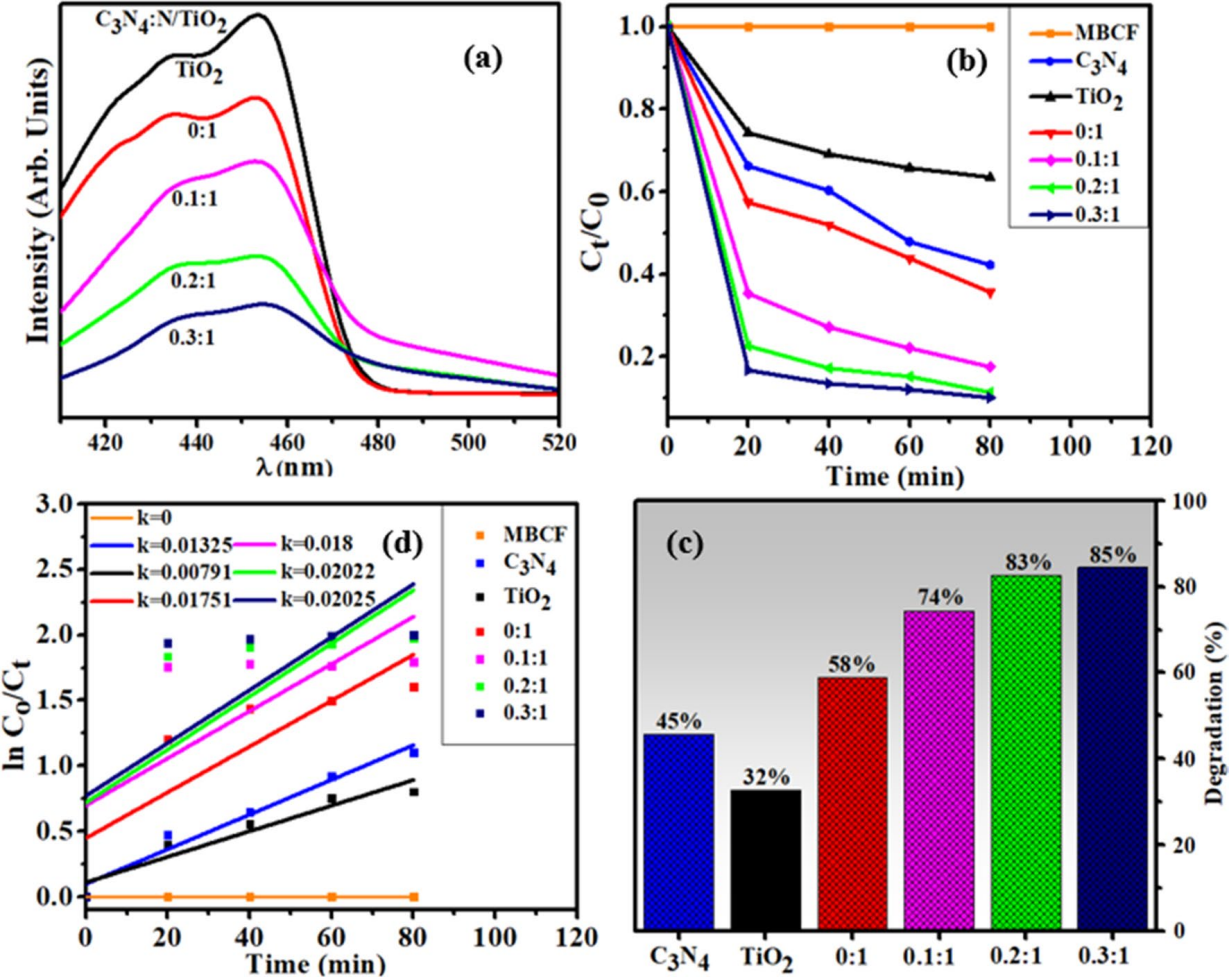
**a** PL spectra (**b**) Plot of concentration ratio (C_t_/C_o_) versus time (**c**) percentage (%) degradation of all samples (**d**) plot of ln(C_o_/C_t_) versus time spectra for dye reduction

PL emission spectra of TiO_2_ and its composites were computed from 410 to 520 nm with an excited wavelength of 350 nm at room temperature, as illustrated in Fig. 6a. Spectra unveiled migration and electron–hole (e^−^ to h^+^) pairs recombination efficiency [40]. Characteristic peak of TiO_2_ at 455 nm showed the highest recombination of e^−^ to h^+^ pairs which significantly limited PCA of TiO_2_ whereas recombination rate was decreased upon co-doping (N and C_3_N_4_). Shockley–Read–Hall (SRH) process explains bandgap transitions from valence band to sub-band and then to conduction band [41]. Sub-band at the edge of conduction band facilitated PCA [42], so for higher doping sample, lower recombination rate proposed higher photo-generated charge transportation that internally enhanced PCA of 0.3:1.

### Reaction Mechanism and Kinetics

**Fig. 7:**
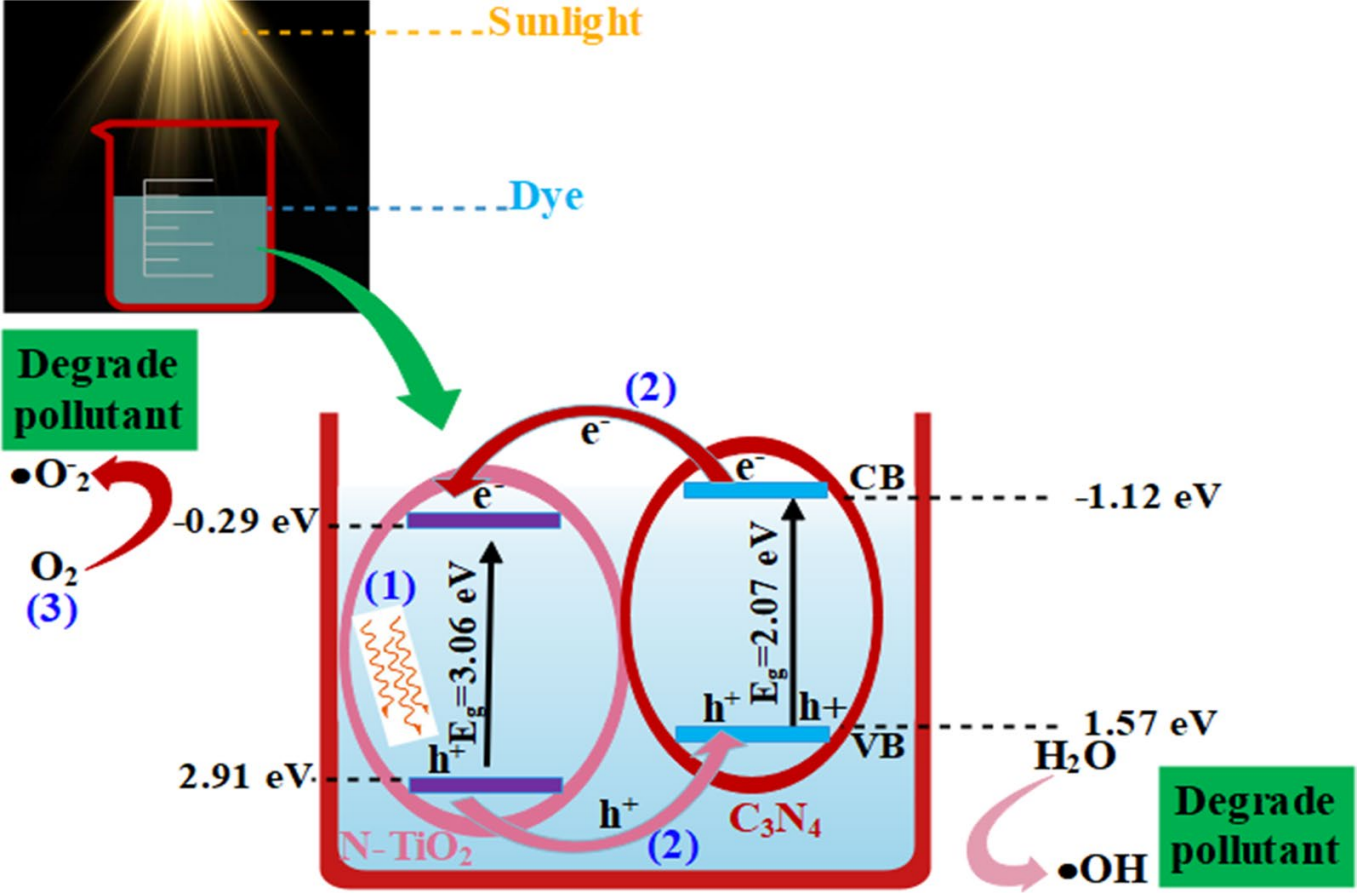
Mechanism for degradation of dyes in the presence of prepared photocatalyst

The following mechanism was involved in PCA of prepared catalyst (see Fig. 7):

1. Photoexcitation: The PCA first involves photoexcitation that initiates by the photons processing equal or greater energy than bandgap energy (E_g_) of material. These photons stimulate electrons of valence/lower band (VB) and migrate them to conduction/higher band (CB). Electrons leave holes behind in VB resulting in e^−^-h^+^ pairs generation, as shown in equation below.
  2 $${\text{TiO}}_{2} + h\nu \to {\text{TiO}}_{2} \left( {e^{ - } } \right) + h^{ + }$$
2. Ionization of water: Holes create OH^•^ free radicals after coming into contact with water (H_2_O).
  3 $${\text{H}}_{2} {\text{O}} + h^{ + } \to {\text{OH}}^{ \cdot } + {\text{H}}^{ + }$$
  The OH^•^ radical act as an oxidizing agent on the surface of semiconductor that targets adsorbed molecules and takes part in mineralization.
3. Oxygen ionosorption: Photogenerated es^−^ comes into contact with water molecules and generate OH^−^ (hydroxyl group) while es^−^ are trapped by molecules of O_2_ to produce O_2_^•˗^ (superoxide radical) [43].
  4 $${\text{O}}_{{2}} + {\text{e}}^{ - } \to {\text{O}}_{{2}}^{ \cdot - }$$
  The superoxide radical contributes in oxidation cycles and inhibits the recombination of e^−^ and h^+^ while keeping the TiO_2_ neutral.
4. Superoxide protonation: Superoxide ions (O_2_^¯^) gives H_2_O^•^ (protonated hydroperoxylate radical) and finally H_2_O_2_ generate OH^•^ radical that is highly reactive.
  5 $${\text{O}}_{{2}}^{ \cdot - } + {\text{ H}} \rightleftharpoons {\text{HOO}}^{ \cdot }$$
  6 $${\text{2HOO}}^{ \cdot } \to {\text{H}}_{{2}} {\text{O}}_{{2}} + {\text{O}}_{{2}}$$
  7 $${\text{H}}_{{2}} {\text{O}}_{{2}} \to {\text{2OH}}^{ \cdot }$$
  8 $${\text{Dye}}\, \, ({\text{MB}}\,{\text{ and}}\,{\text{ CF}}) + {\text{OH}}^{ \cdot } \to {\text{CO}}_{{2}} + {\text{H}}_{{2}} {\text{O }}\left( {{\text{dye}}\,{\text{ intermediates}}} \right)$$
  9 $${\text{Dye}} + {\text{h}}^{ + } \left( {{\text{VB}}} \right) \to {\text{oxidation}}\,{\text{ products}}$$
  10 $${\text{Dye}} + {\text{e}}^{ - } \left( {{\text{CB}}} \right) \to {\text{reduction}}\,{\text{ products}}$$

Oxidation/reduction reactions occurred on photo-excited photocatalysts surface [44, 45].

The PCA of as-prepared samples was evaluated for degradation of MB and CF dye under replicated visible light irradiation (Fig. 6b). The blank test demonstrated that MB and CF could not be degraded under irradiation of light in the absence of catalyst thus it can be deduced that MB and CF were stable. As indicated in Fig. 6c, C3N4 degraded the targeted dye up to 45% and enhanced photocatalytic activity of nanostructures was observed with increasing C_3_N_4_ concentration which effectively degraded MB and CF. In case of N-TiO_2_, relatively higher extent of degradation (58%) occurred as compared to TiO_2_ (32%) while highest doped sample (0.3:1) showed maximum degradation of 85% within 80 min. The apparent reaction rate constants (*k*) were determined for all specimens by measuring slopes of ln (*C*_o_/*C*_t_) against time plot. Moreover, *k* value of 0.3:1 was also higher than others, which was ~ 2.5 times higher than pristine TiO_2_ (Fig. 6d).

The enhanced PCA of nanostructures may be attributed to these measures: firstly, the C_3_N_4_ sheet has a larger surface that encouraged broad adsorption within catalyst and furnished additional active sites for surrounding reactants. Secondly, after incorporation of N and C_3_N_4_ in TiO_2_, increased charge separation efficiency by inducing new energy levels within the forbidden band gap of TiO_2_. These induced levels act as trapping sites for photo induced electrons increasing the electron transfer efficiency which ultimately improved the degradation performance of nano-catalysts. Thirdly, due to intimate and well-matched band edge, N-TiO_2_ collects photo-induced electrons from the CB of C_3_N_4_ thus improving the charge separation efficiency. In general, C_3_N_4_ produces e^−^ to h^+^ pairs under visible light that quickly recombine and only a small fraction of e^−^ participate in PCA. Whereas when TiO_2_ was modified by binary dopants to form a nanocomposite, photo-generated electrons in CB of C_3_N_4_ can directly move to CB of N-TiO_2_ composite, as shown in Fig. 7, because CB edge of C_3_N_4_ was more negative than N-TiO_2_. The above-mentioned parameters eventually increased overall photocatalytic activity of as-prepared nano-composites [46–48].

**Fig. 8:**
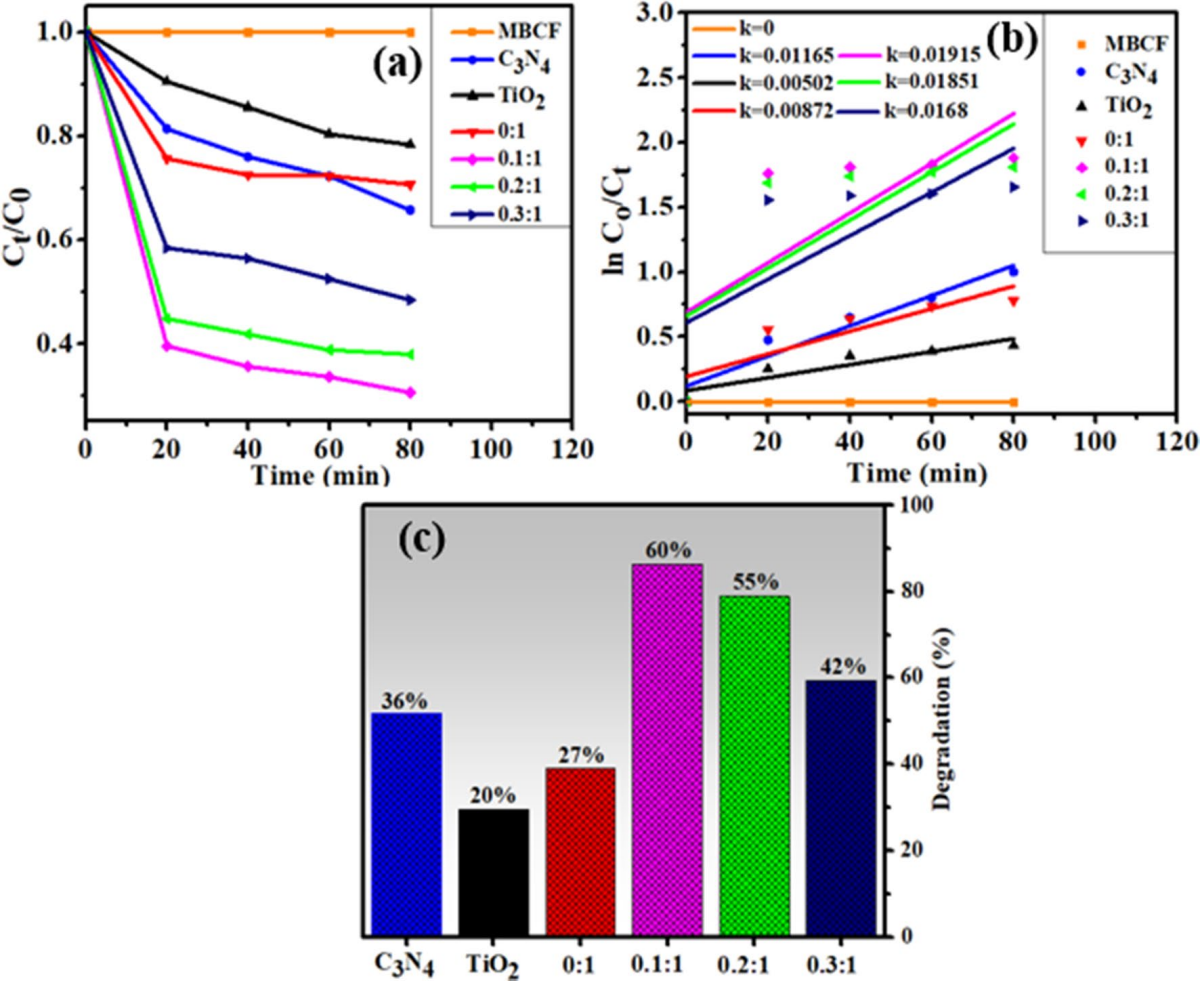
**a** Plot of concentration ratio (*C*_t_/*C*_o_) versus time, **b** plot of ln(*C*_o_/*C*_t_) versus time spectra for dye reduction, **c** percentage (%) degradation of prepared specimens

Sonocatalytic activity (SCA) was also measured by degrading MB and CF via ultra-sonication route. Same as for PCA, samples were collected after 20 min interval in SCA. As illustrated in Fig. 8c, C3N4, TiO_2_, N-TiO_2_ composites degraded the MB and CF up to 36%, 20% and 27% in 80 min while for lower doping, activity of 0.1:1 nanostructure increased up to 60% and then decreased for further and higher doping concentrations. For lower doping, catalyst formed the microbubbles and bore more active sites for growth of nucleation, further generated more reactive radicals [49] and for further doping, active sites of prepared catalysts were insufficient to be occupied by dye molecules. Second possible reason could be surplus of C_3_N_4_ amount that have restricted the energy obtained from ultrasound wave [50].

The rate constants (*k*) have been calculated for sono-degradation kinetics by measuring slopes on ln (*C*_o_/*C*_t_) against time as shown in Fig. 8b. PCA and SCA of pristine TiO_2_ and C_3_N_4_ were less efficient, thus N/C_3_N_4_-doped TiO_2_ composite turned up as potential catalyst for dye degradation.

**Fig. 9:**
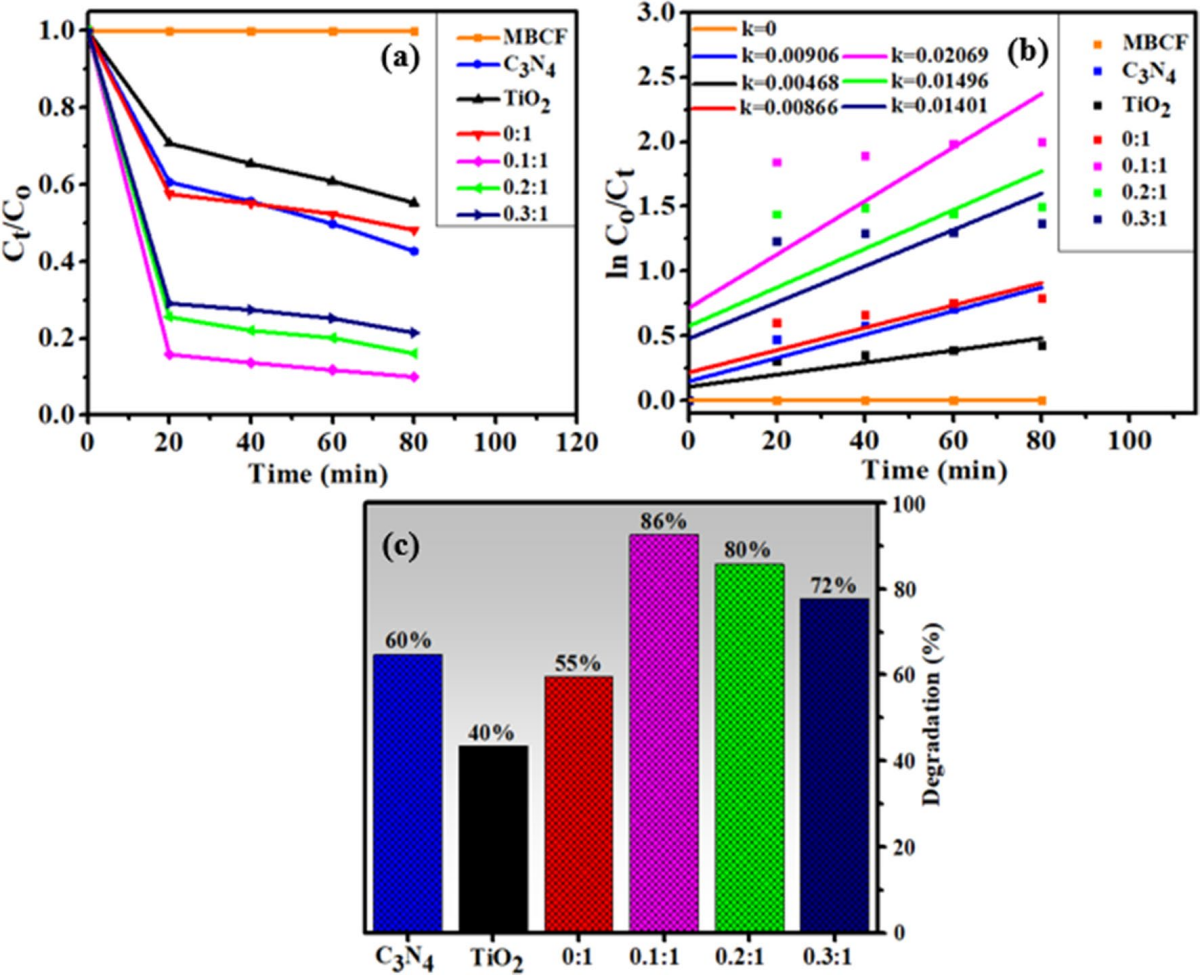
**a** Plot of concentration ratio (*C*_t_/*C*_o_) versus time, **b** plot of ln(*C*_o_/*C*_t_) versus time spectra for dye reduction, **c** percentage (%) degradation of all prepared specimens

The combined effect of PCA and SCA has been evaluated further by adjusting sonometer under light source for all synthesized samples. The earned results unveiled that pristine C_3_N_4_, TiO_2_ and N-TiO_2_ composite degraded dye up to 60%, 40% and 55%, respectively. For lower doping, 0.1:1 nanostructure degraded MB and CF up to 86% caused by combined effect of PCA and SCA. But for further and higher doping (although assisted by PCA) active sites were insufficient that might be ascribed to dominant effect of SCA that subsequently decreased the degradation performance. Also, surplus amount of C_3_N_4_ and inhomogeneous mixing of catalysts can cause agglomeration which might limit the energy obtained from ultrasound wave and visible light source (Fig. 9c).

For photo-sono degradation kinetics, the rate constants (*k*) have been estimated by computing slopes on ln (*C*_o_/*C*_t_) against time, as manifested in Fig. 9b.

Antioxidant characteristics of compounds is tied to their electron or hydrogen atom donating capability to DPPH free radical, such that they create stable diamagnetic compounds [51]. This DPPH free radical’s reduction capability can be examined by lowering the absorbance at 517 nm.

**Fig. 10:**
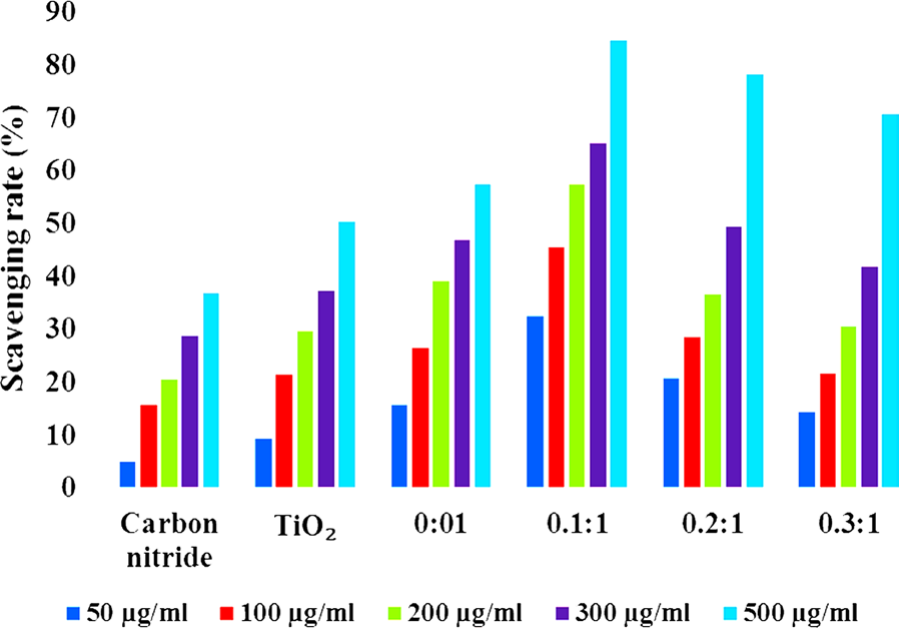
DPPH radical scavenging activity of synthesized nanostructures

All synthesized compounds were evaluated for antioxidant activity using a DPPH radical scavenging assay. Using ascorbic acid as a reference, DPPH disappearance was evaluated spectrophotometrically at 517 nm. In this study, it was discovered that the DPPH activity of the nanoparticles increased in a dose-dependent manner (Fig. 10). It is confirmed that Pristine TiO_2_ showed high scavenging activity (50.22%) at concentration of 500 µg/mL compare to C_3_N_4_. Because TiO_2_ may form OH^.^, O_2_^.−^ and ^1^O_2_ reactive oxygen species, which have the potential to bond with the DPPH free radical [51–53]. Some recent studies have reported ^1^O_2_ to be the dominant active specie in the degradation of MB dye under solar irradiation [53, 54]. While N-TiO_2_ showed DPPH scavenging up to 57.34% that is 7% higher than that of TiO_2_. This considerable increase is resulted from the addition of a doping agent which lowers the size of TiO_2_ nanoparticles and increases their reactivity [55, 56]. In case of C_3_N_4_ doped TiO2 with mass ratio of 0.1:1, scavenging activity increased up to 84.45% that might be the availability of sufficient amount of nitrogen from doped C_3_N_4_. But increasing the concentration of doped C_3_N_4_ on N-TiO_2_, scavenging activity was decreased. This was due to high C_3_N_4_ concentration caused an increase in turbidity of test sample, which in turn caused an antagonistic interaction resulted in a decrease scavenging activity (84.45–70.75%) [50].

**Fig. 11:**
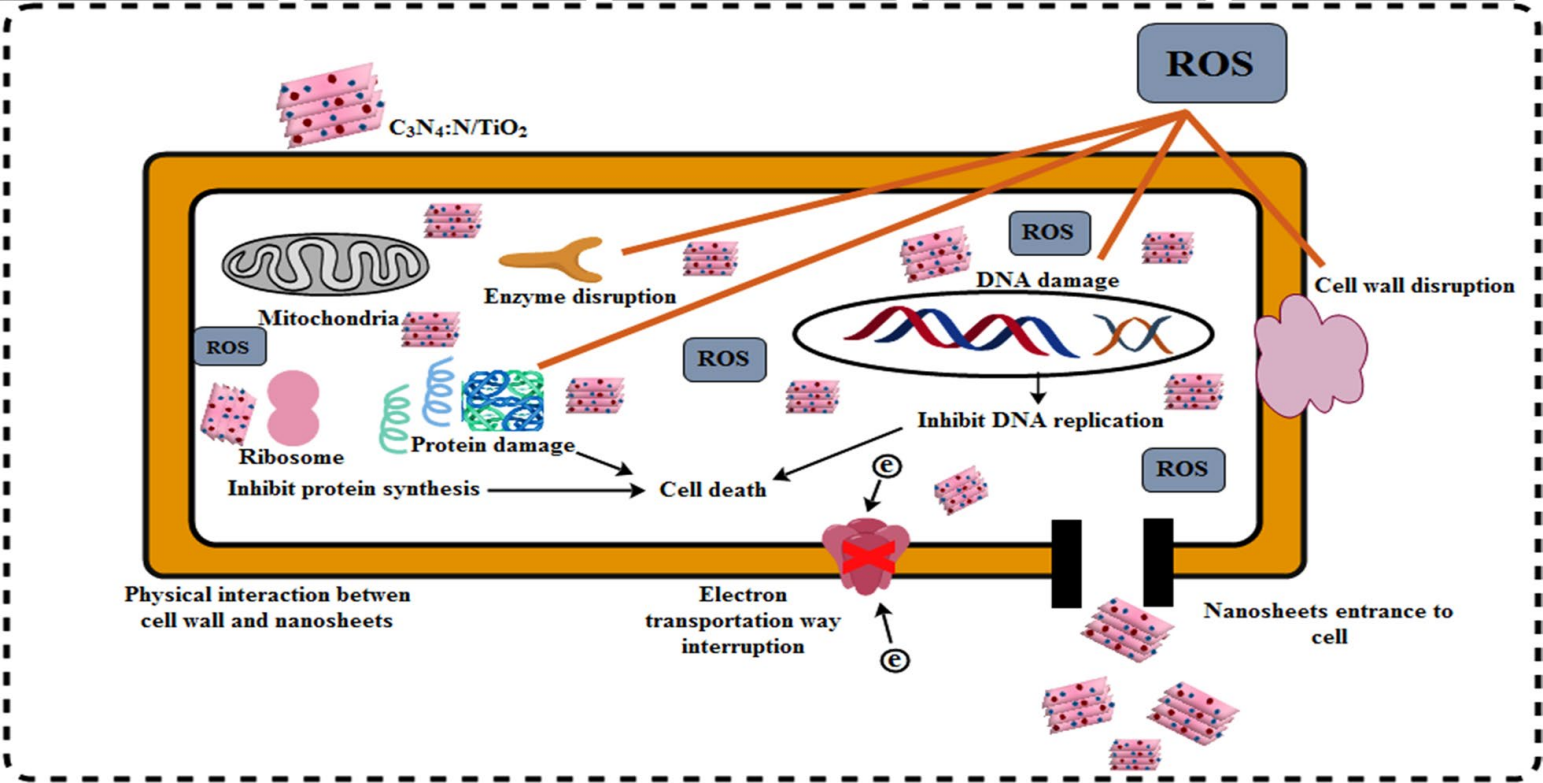
Illustration of antimicrobial activity of prepared sample

Antimicrobial activity of binary doped TiO_2_ was conducted using well diffusion technique (Fig. 11) against *S. aureus* and *E. coli* as depicted in Table 1. Statistically, significant inhibition areas (*p* < 0.05) for minimum and maximum concentrations of doped nanostructures, respectively against *Escherichia coli* (1.05–2.00 mm) and (1.35–2.25 mm) were attained. Broadly, zero activity was observed for TiO_2_ and N-TiO_2_ against *Staphylococcus aureus* at minimum and maximum concentrations while binary-doped samples showed substantial activity against *Escherichia coli* at both concentrations. Similarly, C_3_N_4_ depicted 1.60 mm inhibition area at maximum concentration only against *Escherichia coli*.

**Table 1:** ^a^Inhibition zone (mm) of binary-doped TiO_2_ for *Staphylococcus aureus*. ^b^Inhibition zone determination of binary-doped TiO_2_ for *Escherichia coli*

	Inhibition zone^a^	(mm)	Inhibition zone^b^	(mm)
Sample	0.5 mg/50 µL	1.0 mg/50 µL	0.5 mg/50 µL	1.0 mg/50 µL
TiO_2_	0	0	0	0
N-TiO_2_	0	0	0	0
C_3_N_4_	0	0	0	1.60
0.1:1	0	0	1.05	1.35
0.2:1	0	0	1.50	2.05
0.3:1	0	0	2.0	2.25
Ciprofloxacin	4.45	4.45	4.25	4.25
DIW	0	0	0	0

Anti-bacterial effectiveness is swayed by the scale of nanoparticles so oxidative stress of invented nanocomposites is dependent on scale and concentration [57]. An electrostatic contact between bacteria and nanoscale structures results in the generation of reactive oxygen species, which are lethal to cells [24, 58]. Oxygen reactive species (ROS) encircle bacteria external membrane and through extrusion and bulge of cytoplasmic components bacteria death occurs [59]. Micro pathogens ruin also proceeds when cations strongly bind with negative components of bacterial cells. Cations cause dysfunction in bacterial ribosomal activities and enzymatic degradation resulting collapse [60]. Two reactions have been identified as feasible for the bactericidal mechanism of nanomaterials, one of which involves strong interaction between the cations Ti^+4^ and bacterial cells, resulting in the formation of negativized sections and subsequent collapse, and the other of which involves electronic excitation of the TiO_2_ valance band surface via irradiation. Additionally, the electrical O_2_ reaction generates O^−2^ radicals, which results in the production of H_2_O_2_. The resultant O^−2^ species play a critical role in the breakdown of lipid or protein molecules on the bacteria's external cell membrane [61, 62].

## Conclusion

Binary-doped TiO_2_ was synthesized through co-precipitation method and synthesized samples were evaluated for photo, sono and photo-sono catalytic degradation of MB and CF dyes and bactericidal activities. The strong contact formation between dopants and TiO_2_ efficiently increased e^−^ to h^+^ pairs separation efficiency induced by light. The narrow bandgap of C_3_N_4_:N/TiO_2_ composite was accredited to N as well as C_3_N_4_ incorporation in pristine TiO_2_. The prepared samples showed efficient degradation performance under visible light as well as under ultrasonic waves (SCA). Moreover, the combined effect of photo and sono catalysis was also evaluated for prepared catalysts for comparative study. Furthermore, prepared nanocomposites exhibited notable efficacy against *S. aureus* and *E. coli* bacteria as well. We believe that this study will open new insights into the fabrication of novel, binary doped heterojunctions for effective dye degradation and bactericidal applications in the future.

## Data Availability

All data are fully available without restriction.

## Abbreviations

C_3_N_4_: Carbon nitride
EDS: Energy dispersive X-ray spectroscopy
FTIR: Fourier transform infrared spectroscopy
FESEM: Field emission scanning electron microscopy
G + ve: Gram-positive
G −ve: Gram negative
GO: Graphene
HR-TEM: High resolution transmission electron microscopy
JCPDS: Joint committee on powder diffraction standards
TiO_2_: Titanium dioxide
UV–vis: Ultra-violet visible spectroscopy
XRD: X-ray diffraction
